# m^6^A RNA modification and myeloid-derived suppressor cells: mechanistic insights and clinical prospects

**DOI:** 10.3389/fimmu.2026.1694842

**Published:** 2026-01-23

**Authors:** Chunhong Li, Xiulin Jiang, Yixiao Yuan, Xi Chen, Shanrui Pu, Kun Lian, Lihua Li, Qiang Wang

**Affiliations:** 1 Department of Oncology, Suining Central Hospital, Suining, Sichuan, China; 2 Department of Systems Biology, City of Hope Comprehensive Cancer Center, Monrovia, CA, United States; 3 Biomedical Research Center, Monrovia, CA, United States; 4 Kunming Medical University, Kunming, China; 5 School of Biosciences, University of Birmingham, Birmingham, United Kingdom; 6 Department of Gastrointestinal Surgical Unit, Suining Central Hospital, Suining, Sichuan, China

**Keywords:** epitranscriptome, immunotherapy, m6A modification, MDSC, tumor microenvironment

## Abstract

N6-methyladenosine (m^6^A) is the most abundant post-transcriptional modification in eukaryotic mRNA, extensively involved in RNA splicing, export, stability, and translation. In recent years, accumulating evidence has demonstrated that m^6^A modification plays a critical role in regulating the differentiation and function of immune cells. Among these, myeloid-derived suppressor cells (MDSCs), as a key immunosuppressive population within the tumor microenvironment (TME), accelerate tumor progression by inhibiting T cell activity and promoting immune evasion and therapy resistance. Emerging studies indicate that m^6^A modification modulates the development, accumulation, and immunosuppressive function of MDSCs, thereby contributing to tumor initiation and progression. This review provides a narrative overview of the current evidence regarding the crosstalk between m^6^A modification and MDSCs, with a focus on the underlying molecular mechanisms and their potential implications for cancer immunotherapy. Furthermore, we discuss future research directions and the challenges associated with clinical translation.

## 1 Introduction

M^6^A is the most prevalent internal modification of eukaryotic messenger RNA (mRNA) and has emerged as a crucial layer of epitranscriptomic regulation (1, 2). By dynamically modulating RNA splicing, stability, nuclear export, and translation, m^6^A modification fine-tunes gene expression at the post-transcriptional level and exerts profound impacts on diverse biological processes, including cell differentiation, stress responses, and immune regulation (3–6).

MDSCs are a heterogeneous population of immature myeloid cells that expand in pathological conditions such as cancer, chronic infection, and inflammation (7–9). Within the tumor microenvironment, MDSCs play a pivotal role in promoting immune suppression, facilitating tumor progression, and contributing to therapeutic resistance (10–12). They exert their immunosuppressive functions through multiple mechanisms, including inhibition of T-cell proliferation and activation, induction of regulatory T cells, and modulation of other immune and stromal components (13).

Recent studies have begun to reveal a close interplay between RNA m^6^A modification and MDSC biology. m^6^A regulators have been shown to influence the differentiation, expansion, and immunosuppressive functions of MDSCs, thereby shaping the immune landscape of tumors (14–17). Understanding the m^6^A-MDSC axis not only provides new mechanistic insights into tumor immune evasion but also opens up potential therapeutic opportunities for targeting MDSCs in cancer and other immune-related diseases. In this review, we summarize current knowledge on the roles of m^6^A methylation in MDSC regulation, highlight their functional consequences in the tumor microenvironment, and discuss therapeutic implications and future perspectives.

## 2 Overview of RNA m^6^A methylation

**Figure 1:**
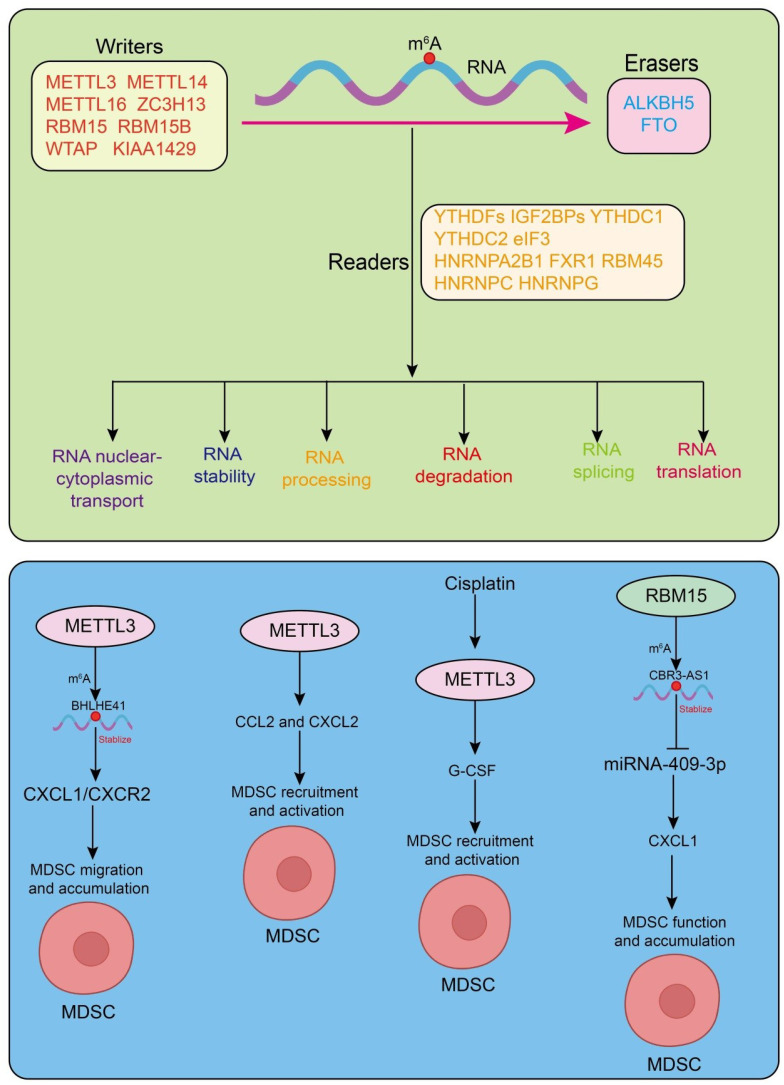
Dynamic regulation and functions of m^6^A modification. Upper panel:Schematic representation of the m^6^A RNA methylation process, including the coordinated action of methyltransferases (writers), demethylases (erasers), and m^6^A-binding proteins (readers), and their roles in post-transcriptional gene regulation. Lower panel: overview of how m^6^A “writers” regulate the expansion, differentiation, and immunosuppressive function of MDSCs.

m^6^A is dynamically regulated by a set of reversible factors. The core regulatory network of m^6^A consists of “writers, “ “erasers, “ and “readers.” Writers are responsible for catalyzing m^6^A deposition. METTL3 and METTL14 form the catalytic core, while WTAP acts as a scaffold to stabilize the complex and recruit substrate RNAs (16, 18–23). RBM15 and RBM15B guide m^6^A deposition by binding specific RNA regions, and METTL16 mediates m^6^A modification on select RNA substrates (24–26). Erasers confer reversibility to m^6^A through demethylation, with FTO and ALKBH5 being the primary enzymes. FTO was the first identified m^6^A demethylase and is involved in metabolic regulation and tumorigenesis, whereas ALKBH5 plays crucial roles in RNA stability, cell proliferation, and modulation of the tumor immune microenvironment (27–30). Readers recognize and bind m^6^A-modified sites, thereby determining the fate of target RNAs. The YTH domain family proteins (YTHDF1/2/3, YTHDC1/2) modulate mRNA translation, stability, and nuclear export (31–35). IGF2BP family members (IGF2BP1/2/3) stabilize m^6^A-modified transcripts to enhance their expression (31, 36). Heterogeneous nuclear ribonucleoproteins (HNRNPs), including HNRNPA2B1 (37), influence alternative splicing and pre-mRNA processing, while other m^6^A-binding proteins such as FXR1, RBM45, and RBM33 also participate in RNA metabolism and gene regulation (38–41). Functionally, m^6^A modification fine-tunes gene expression and broadly participates in immune regulation and tumorigenesis. In the immune system, m^6^A modulates the differentiation and function of dendritic cells, T cells, and macrophages, thereby affecting antigen presentation and immune responses. Notably, its regulation of immunosuppressive cells, including MDSCs, plays a pivotal role in shaping the immunosuppressive TME (**Figure 1**).

## 3 MDSCs: biology and immunosuppressive functions

MDSCs originating from the bone marrow (8, 9). Under normal physiological conditions, myeloid progenitors gradually differentiate into mature neutrophils, monocytes, macrophages, or dendritic cells (42). However, in pathological states such as cancer, chronic inflammation, infection, and autoimmune diseases, myeloid differentiation is disrupted, leading to the accumulation of immature immunosuppressive cells that constitute MDSCs (41, 43, 44).

MDSCs consist of heterogeneous subsets with distinct developmental origins, phenotypes, and suppressive mechanisms. PMN-MDSCs (polymorphonuclear MDSCs) represent a population closely resembling neutrophils, characterized by CD11b^+^Ly6G^+^Ly6C^low^ in mice and CD11b^+^CD15^+^/CD66b^+^HLA-DR^-^ in humans (45). They primarily suppress T-cell responses through reactive oxygen species (ROS), Arg1, and other oxidative pathways. In contrast, M-MDSCs (monocytic MDSCs) resemble monocytes and are defined by CD11b^+^Ly6C^high^Ly6G^-^ in mice and CD11b^+^CD14^+^HLA-DR^-^ in humans. They typically exert immunosuppression through iNOS/NO, COX2-PGE2 signaling, and metabolic reprogramming. A third subset, eMDSCs (early-stage MDSCs), consists of immature myeloid progenitors that lack clear neutrophil or monocyte lineage markers. They are identified in humans as Lin^-^HLA-DR^-^CD33^+^ cells and represent a more primitive suppressive population involved in early immune dysregulation. Together, these three MDSC subsets differ in lineage characteristics, phenotype, and suppressive mechanisms, reflecting their diverse roles across inflammatory, autoimmune, and tumor settings (46).

The expansion and functional activation of MDSCs are tightly regulated by multiple signaling pathways, among which STAT3 is considered a central driver (47, 48). Cytokines such as IL-6, G-CSF, and GM-CSF activate STAT3 in various tumor contexts, promoting MDSC survival, blocking their differentiation, and inducing the expression of immunosuppressive molecules, including arginase-1 (ARG1) and inducible nitric oxide synthase (iNOS) (49–51). The NF-κB pathway further enhances MDSC suppressive activity through upregulation of inflammatory cytokines such as IL-1β and TNF-α (52). Within hypoxic tumor microenvironments, HIF-1α signaling promotes the differentiation of M-MDSCs into tumor-associated macrophages (TAMs) and drives the expression of lactate and ARG1, thereby inhibiting T cell effector function (45, 53, 54). Transcription factors such as C/EBPβ also play essential roles in mediating myeloid differentiation arrest and regulating immunosuppressive genes (55, 56). Moreover, MDSCs undergo extensive metabolic reprogramming, with enhanced glycolysis, fatty acid oxidation, and glutamine metabolism sustaining both cellular energy demands and the production of immunosuppressive mediators.

MDSCs suppress immune responses through multiple mechanisms. First, high expression of ARG1, iNOS, and indoleamine 2, 3-dioxygenase (IDO) depletes L-arginine and tryptophan in the microenvironment, limiting T cell proliferation and function (57). Second, reactive oxygen species (ROS) and reactive nitrogen species (RNS) directly damage the T cell receptor (TCR-CD3) complex, impairing antigen recognition (58). Third, upregulation of immune checkpoint molecules, such as PD-L1, promotes T cell exhaustion. Fourth, MDSCs secrete cytokines including IL-10 and TGF-β to expand regulatory T cells (Tregs), indirectly reinforcing immunosuppressive effects (50). Additionally, they inhibit natural killer (NK) cell cytotoxicity and impair dendritic cell-mediated antigen presentation. MDSCs play pivotal roles in various pathological conditions. In cancer, they accumulate in peripheral blood and the tumor microenvironment, suppress anti-tumor T cell and NK cell responses, promote angiogenesis, invasion, and metastasis, and are closely associated with resistance to immunotherapy (11, 45, 59). In chronic infections, MDSCs suppress excessive immune responses, mitigating tissue damage but potentially allowing pathogen persistence (60, 61). In chronic inflammatory and autoimmune diseases, such as rheumatoid arthritis and inflammatory bowel disease, MDSC accumulation can alleviate inflammation, yet excessive immunosuppression may impair immune surveillance (62–64). In summary, MDSCs are highly heterogeneous immunosuppressive cells that not only play critical roles in tumor development but also broadly regulate immune responses in infection and inflammatory diseases. Therefore, a deeper understanding of MDSC biology and molecular mechanisms is essential for developing novel immunomodulatory and anti-tumor therapeutic strategies.

## 4 Crosstalk between m^6^A methylation and MDSCs

To provide a comprehensive overview of current research, we summarized the major m6A regulators, the experimental models used, their functions in MDSCs, associated mechanisms, and key references in **Table 1**.

**Table 1:** Key m6A regulators and their roles in MDSC biology across experimental models.

m6A regulator	Experimental model	Function	Mechanism	References
METTL3	CRC murine models (CT26, MC38)	Promotes MDSC accumulation; suppresses CD4^+^/CD8^+^ T-cell activity	METTL3 ↑ → BHLHE41 ↑ → CXCL1 ↑ → CXCL1/CXCR2-dependent MDSC migration	(66)
METTL3	Ovarian cancer model with Mettl3 conditional knockout (cKO) mice	Mettl3 deficiency enhances tumor growth; increases CCL2/CXCL2 and recruitment of immunosuppressive myeloid cells (including MDSCs)	Mettl3 loss increases IL-1β secretion (inflammasome-independent), promoting immunosuppressive myeloid activation	(15)
METTL3	Human bladder cancer, cisplatin IAIC therapy	METTL3 inhibition decreases G-CSF and reduces fibrotic MDSC (f-MDSC) accumulation	Cisplatin ↓ METTL3 → ↓ G-CSF production → impaired MDSC expansion and function	(67)
RBM15	Radioresistant NSCLC (clinical samples + *in vivo* murine models)	Enhances MDSC recruitment; correlates with poor prognosis; silencing RBM15 reduces MDSC infiltration and restores CD4^+^/CD8^+^ T-cell immunity	RBM15 → m6A-IGF2BP3-dependent upregulation of CBR3-AS1 → sponging miR-409-3p → CXCL1 upregulation → MDSC recruitment	(68)
ALKBH5	ICB treatment murine model	ALKBH5 deletion enhances ICB efficacy; reduces MDSC-mediated immunosuppression	Regulates m6A-dependent expression of Mct4/Slc16a3 → alters lactate levels → affects Tregs and MDSCs	(69).
ALKBH5	CRC murine model	High ALKBH5 promotes MDSC accumulation; reduces CD8^+^ T cells and NK cells	Demethylation of AXIN2 → reduced IGF2BP1 binding → AXIN2 degradation → activation of Wnt/β-catenin–DKK1 axis → MDSC recruitment	(70).
ALKBH5	ALKBH5 cKO mice	Regulates the immunosuppressive activity of MDSCs	ALKBH5 modulates m6A on Arg-1 mRNA → reduced stability → decreased MDSC suppressive function	(71)
ALKBH5	SLE model	ALKBH5 in M-MDSCs ameliorates autoimmunity by regulating B-cell activation	ALKBH5 → m6A-dependent upregulation of FoxO1 → suppression of Met/COX2/PGE2 pathway	(72)
YTHDF1	CRC murine models (CT26, MC38)	Ythdf1 deletion reduces MDSC abundance and enhances anti-tumor immunity; overexpression promotes immunosuppressive TIME	YTHDF1 regulates p65–CXCL1 axis → MDSC migration → CD8^+^ T-cell suppression	(14).
YTHDF1	NASH-HCC murine model	Promotes MDSC recruitment and activation; drives spontaneous NASH-HCC development	YTHDF1 regulates EZH2–IL-6 axis → immunosuppressive microenvironment	(73)
YTHDF2	Ionizing radiation (IR) models, mouse + human samples	IR induces YTHDF2 → expands immunosuppressive MDSCs; Ythdf2 deficiency reverses radioresistance	NF-κB–dependent YTHDF2 induction; YTHDF2 degrades NF-κB negative regulators (positive feedback loop)	(74)
YTHDF2	Ythdf2 cKO mice	Regulates MDSC infiltration and suppressive activity	m6A-dependent degradation of Bambi mRNA	(75)
YTHDF2	HCC murine model with pH-responsive Lip@si-YTHDF2 nanoparticles	YTHDF2 inhibition reduces immunosuppressive MDSC phenotype and restores T-cell immunity	YTHDF2 inhibition → m6A-mediated degradation of MYC → restored anti-tumor immunity	(76)
YTHDF2	Ythdf2 cKO mice	cKO causes progressive accumulation of MDSCs (especially PMN-MDSCs) in liver → bone marrow; enhances suppressive activity	Identified RXRα as a direct m6A target of YTHDF2; YTHDF2 promotes RXRα mRNA degradation	(77)
HNRNPA2B1	Human CRC → liver metastasis (CRLM) patient samples & EV mouse CRC models	Indirectly promotes MDSC recruitment via liver stromal remodeling	HNRNPA2B1 loads MIR181A1HG into EVs → ceRNA activity → inhibits miR-373-3p → activates hepatic stellate cells → CXCL12 secretion → MDSC recruitment	(78)

### 4.1 Functions and mechanisms of m^6^A methyltransferases in MDSCs

Current studies increasingly support the view that m^6^A RNA methylation plays a critical role in the tumor immune microenvironment by regulating MDSCs (65). In colorectal cancer (CRC) murine models, knockdown of METTL3 in CRC cells markedly reduced MDSC accumulation, thereby maintaining the activation and proliferation of CD4^+^ and CD8^+^ T cells and ultimately inhibiting tumor growth. Mechanistically, METTL3 promotes BHLHE41 expression in an m^6^A-dependent manner, which in turn induces CXCL1 transcription, enhancing MDSC migration (66). *In vitro* experiments further showed that silencing BHLHE41 or treating with CXCL1 protein/CXCR2 inhibitor SB265610 significantly attenuated METTL3-mediated MDSC migration, indicating that this effect primarily depends on the BHLHE41–CXCL1/CXCR2 axis. Moreover, *in vivo* experiments revealed that depletion of MDSCs using anti-Gr1 antibodies or SB265610 effectively blocked the tumor-promoting effect of METTL3 (66). Similarly, in ovarian cancer (OC) models, OC cells establish an immunosuppressive microenvironment by altering host immune cell functions. In Mettl3 conditional knockout (cKO) mice, OC cell growth was significantly enhanced, and Mettl3 deficiency in myeloid cells increased CCL2 and CXCL2 secretion in the peritoneal lavage fluid, thereby promoting recruitment and activation of immunosuppressive myeloid cells, such as MDSCs (15). Interestingly, Mettl3 deletion also augmented IL-1β secretion induced by surviving OC cells, independently of inflammasome activation and cell death (15). In human bladder cancer, cisplatin administered via intra-arterial infusion chemotherapy (IAIC) similarly inhibited METTL3 activity in tumor cells, reducing granulocyte colony-stimulating factor (G-CSF) levels, which correlated with decreased m^6^A methylation (67). Cisplatin-mediated reduction of METTL3 suppressed G-CSF production, thereby inhibiting the accumulation and immunosuppressive function of fibrotic MDSCs (f-MDSCs) and improving the local tumor immune microenvironment (67). Additionally, radiotherapy, a standard treatment for locally advanced human non-small cell lung cancer (NSCLC), is often limited by radioresistance. Clinical studies have shown that RBM15 protein is highly expressed in radioresistant patients and correlates with poor prognosis. Mechanistically, RBM15 upregulates CBR3-AS1 via an m^6^A-IGF2BP3-dependent pathway and promotes CXCL1 expression by sponging miR-409-3p, leading to MDSC recruitment and suppression of T cell activity (68). *In vivo* experiments further demonstrated that RBM15 silencing reduced MDSC infiltration and enhanced CD8^+^ and CD4^+^ T cell tumor infiltration, thereby overcoming NSCLC radioresistance (68) (**Figure 1**). Collectively, these studies consistently indicate that m^6^A methyltransferases, such as METTL3 and RBM15, regulate MDSC recruitment and immunosuppressive functions across multiple tumor types, profoundly influencing tumor growth, metastasis, and therapeutic resistance. Therefore, targeting m^6^A regulatory pathways to inhibit MDSC function, alone or in combination with existing immunotherapy and radio/chemotherapy strategies, may represent a promising approach to enhance antitumor efficacy.

### 4.2 Functions and mechanisms of m^6^A demethylases in MDSCs

**Figure 2:**
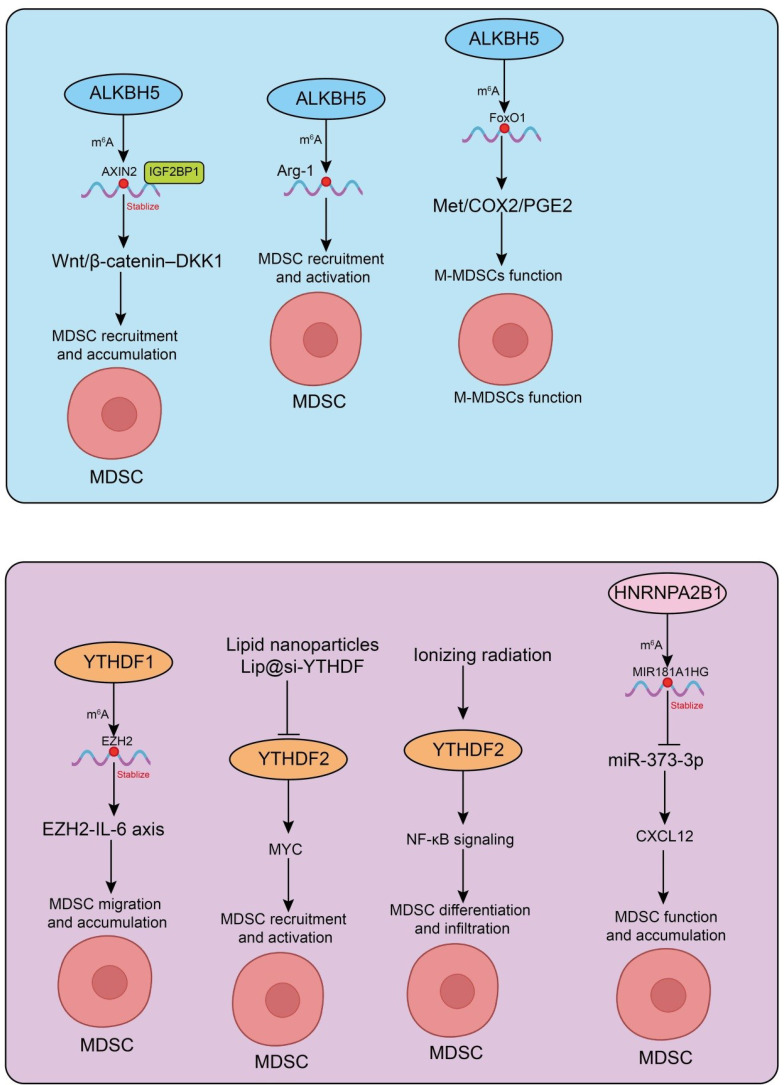
Functions and mechanisms of m^6^A demethylases and m^6^A reader proteins in MDSCs. Depiction of m^6^A “erasers” and m^6^A “readers” interpreting methylation marks in MDSCs, affecting mRNA fate and downstream immunoregulatory functions.

Recent advances have provided evidence that the m6A demethylase ALKBH5 plays a pivotal role in tumor immune regulation. Notably, ALKBH5 deletion has been shown to significantly enhance tumor sensitivity to immune checkpoint blockade (ICB) therapy. During ICB treatment, ALKBH5 modulates m^6^A levels and RNA splicing events in tumor cells, thereby influencing the expression of Mct4/Slc16a3 and lactate content in the tumor microenvironment, which in turn alters the composition of tumor-infiltrating Tregs and MDSCs (69). Specifically, ALKBH5 inhibition reduces MDSC-mediated immunosuppression and enhances the efficacy of immunotherapy, suggesting that ALKBH5 may serve as a potential adjuvant target for immune-based therapies (69). In CRC murine model, high ALKBH5 expression is frequently associated with poor prognosis. Animal studies further indicate that ALKBH5 promotes MDSC accumulation while reducing NK cells and cytotoxic CD8^+^ T cells, thereby facilitating tumor development and immune evasion (70). Mechanistic investigations revealed that ALKBH5 demethylates AXIN2 mRNA, leading to its dissociation from the m^6^A reader IGF2BP1 and subsequent degradation. This process activates the Wnt/β-catenin–DKK1 signaling axis, resulting in MDSC recruitment and suppression of CD8^+^ T cell and NK cell function (70). Targeting ALKBH5 or DKK1 effectively diminishes MDSC accumulation and enhances anti-PD-1 therapeutic responses, highlighting new potential strategies for CRC immunotherapy (70). Additionally, another study demonstrated that ALKBH5 regulates m^6^A modifications of Arg-1 mRNA in MDSCs, reducing its stability and expression, thereby attenuating MDSC immunosuppressive activity and inhibiting tumor progression (71). These findings further underscore the critical role of ALKBH5 in modulating MDSC function and antitumor immunity. Importantly, ALKBH5 also plays a key role in autoimmune disease. In a systemic lupus erythematosus (SLE) model, ALKBH5 upregulates FoxO1 expression in monocytic MDSCs (M-MDSCs) via m^6^A modification, suppresses the Met/COX2/PGE2 signaling pathway, and thereby regulates B cell proliferation and activation, ultimately ameliorating disease progression (72). This discovery reveals the central role of the ALKBH5-FoxO1-M-MDSC axis in immune regulation and provides a potential therapeutic target for autoimmune interventions (72) (**Figure 2**). Collectively, these studies indicate that ALKBH5 regulates MDSC-mediated immunosuppressive functions through multiple mechanisms in both tumor and autoimmune contexts. As such, targeting ALKBH5 not only represents a promising strategy to enhance cancer immunotherapy but also offers novel insights for the treatment of autoimmune diseases.

### 4.3 Functions and mechanisms of m^6^A reader proteins in MDSCs

Evidence continues to accumulate, indicating that RNA m^6^A modification plays a crucial role in regulating the differentiation, activation, and function of MDSCs within the tumor immune microenvironment. In CRC murine models, Ythdf1 deletion markedly enhances anti-tumor immune responses in CT26 and MC38 syngeneic tumors, whereas Ythdf1 overexpression promotes an immunosuppressive TIME and accelerates CRC progression (14). Single-cell RNA sequencing demonstrates that Ythdf1-deficient tumors exhibit reduced MDSC abundance alongside increased cytotoxic T cells. Mechanistic studies indicate that YTHDF1 regulates the p65-CXCL1-MDSC axis via m^6^A modification, promoting MDSC migration and suppressing CD8^+^ T cell function, thereby shaping an immunosuppressive TIME (14). Targeting YTHDF1 reduces MDSC accumulation and enhances anti-PD-1 therapy efficacy, suggesting a potential therapeutic target for CRC immunotherapy (14). Similarly, in non-alcoholic steatohepatitis-related hepatocellular carcinoma (NASH-HCC) murine model, YTHDF1 is significantly overexpressed in tumor tissues. Liver-specific Ythdf1 overexpression drives spontaneous NASH-HCC development and promotes MDSC recruitment and activation through m^6^A-mediated regulation of the EZH2-IL-6 axis, concurrently suppressing CD8^+^ T cell function and establishing an immunosuppressive TIME (73). Targeting YTHDF1 likewise improves anti-PD-1 therapeutic responses, highlighting a potential immunotherapeutic target in NASH-HCC (73). Moreover, ionizing radiation (IR) has been shown to induce expansion of immunosuppressive MDSCs and upregulate YTHDF2 expression in both mouse models and humans (74). Following IR exposure, Ythdf2 deficiency in myeloid cells enhances anti-tumor immunity and reverses tumor radioresistance by modulating MDSC differentiation, infiltration, and immunosuppressive activity (74). Further studies reveal that IR-induced YTHDF2 expression is dependent on the NF-κB signaling pathway, and YTHDF2 forms a positive feedback loop by directly binding and degrading NF-κB negative regulators. Pharmacological inhibition of YTHDF2 overcomes MDSC-mediated immunosuppression and improves the efficacy of radiotherapy and radiotherapy/anti-PD-L1 combination therapy (74). Additionally, YTHDF2 degrades Bambi mRNA via an m^6^A-dependent mechanism, thereby regulating MDSC infiltration and immunosuppressive function, which affects post-radiotherapy anti-tumor immunity. Targeting BAMBI enhances both radiotherapy and combined radiotherapy/immunotherapy efficacy, representing a novel strategy to overcome tumor radioresistance (75). In HCC murine model, pH-responsive lipid nanoparticles (Lip@si-YTHDF2) effectively deliver siRNA to suppress YTHDF2, reduce the immunosuppressive phenotype of MDSCs, restore T cell-mediated anti-tumor immunity, and significantly inhibit tumor growth in combination with PD-1 blockade, providing a feasible strategy for HCC immunotherapy (76). Mechanistic studies revealed that Lip@si-YTHDF2 inhibits YTHDF2, promotes m^6^A-mediated degradation of MYC, and restores antitumor immunity in hepatocellular carcinoma (76). Notably, tumor-derived extracellular vesicles (EVs) also contribute to MDSC regulation.

In YTHDF2 conditional knock-out mice, the loss of YTHDF2 resulted in a gradual elevation of MDSCs including PMN-MDSCs both in liver and ultimately in the BM (77). Loss of YTHDF2 in myeloid cells leads to a progressive accumulation of MDSCs, particularly PMN-MDSCs, first in the liver and eventually in the bone marrow. These YTHDF2-deficient MDSCs exhibit enhanced suppressive capacity toward T cell proliferation, and their increased expansion and chemotactic migration to the liver contribute to the attenuation of ConA-induced immune-mediated liver injury (77). Mechanistically, multi-omic analyses identified RXRα as a direct m6A-modified target of YTHDF2. YTHDF2 binds RXRα mRNA and promotes its degradation, thereby limiting RXRα expression under physiological conditions (77).

In human colorectal cancer liver metastasis (CRLM), tlhe long non-coding RNA MIR181A1HG is progressively upregulated in patient tissues and serum EVs. HNRNPA2B1 mediates its packaging into CRC-derived EVs, and through a competing endogenous RNA (ceRNA) mechanism, sequesters miR-373-3p, thereby activating hepatic stellate cells (HSCs) (78). Activated HSCs secrete CXCL12, which remodels the extracellular matrix and recruits MDSCs to the liver, promoting hepatic metastasis (**Figure 2**). Collectively, these studies indicate that m^6^A modification regulates MDSC differentiation, recruitment, activation, and immunosuppressive function through multiple layers and pathways. This not only provides new insights into the mechanisms of tumor immune suppression but also identifies potential molecular targets for MDSC-directed immunotherapy. These findings highlight the clinical potential of modulating m^6^A modification to reshape the tumor immune microenvironment.

Across different models, m6A regulation appears to show subtype-specific patterns in MDSCs. In PMN-MDSCs, YTHDF2 exerts a dominant role by binding to and destabilizing RXRα mRNA, thereby restraining PMN-MDSC accumulation and suppressive function (77). Loss of YTHDF2 markedly increases PMN-MDSCs in the liver and bone marrow and enhances their immunosuppressive capacity. In contrast, in M-MDSCs the central axis involves ALKBH5-mediated m6A demethylation of FoxO1 mRNA. Reduced FoxO1, either in lupus patients or in FoxO1-deficient mice, impairs the Met/COX2/PGE2 pathway, leading to B-cell hyperactivation (72). Conversely, upregulation of FoxO1 through ALKBH5 or pharmacological interventions (e.g., SF/DP) increases FoxO1 stability, inhibits glycolysis via PFKFB3, and reinforces the suppressive activity of M-MDSCs. Thus, whereas PMN-MDSCs rely on m6A-dependent degradation driven by YTHDF2, M-MDSCs are controlled by m6A demethylation that stabilizes FoxO1, underscoring distinct epitranscriptomic programs that fine-tune the immunoregulatory functions of these two MDSC subsets.

## 5 Therapeutic implications

With growing insights into the role of m^6^A modification in regulating MDSCs, targeting m^6^A regulators to modulate MDSC accumulation and function has emerged as a promising therapeutic strategy. Current research primarily focuses on modulating the activity of m^6^A “writers, “ “erasers, “ and “readers” to alter the fate of MDSC-associated transcripts, thereby alleviating immunosuppression within the tumor microenvironment (69). First, inhibitors of m^6^A demethylases (“erasers”) have demonstrated significant anti-tumor potential. For example, FTO inhibitors, such as R-2-hydroxyglutarate (R-2HG), can stabilize key anti-tumor transcripts, suppress MDSC-mediated immunosuppressive functions, and enhance T cell-mediated anti-tumor immunity (79, 80). Similarly, ALKBH5 inhibitors have been reported to reduce MDSC accumulation within the TME and decrease the expression of immunosuppressive factors, thereby improving responses to immunotherapy (70). Conversely, modulating the activity of m^6^A methyltransferases (“writers”) also holds therapeutic promise. METTL3, as a core methyltransferase, has been shown to regulate MDSC differentiation and function, and specific activators or inhibitors of METTL3 could potentially be used to precisely manipulate the immunosuppressive activity of MDSCs. Second, combination therapies represent an emerging focus in m^6^A-targeted treatment strategies. Since MDSCs play a critical role in resistance to immune checkpoint inhibitors (ICIs), co-administration of m^6^A-targeted agents with ICIs may provide synergistic benefits. For instance, combining FTO or ALKBH5 inhibitors with anti-PD-1/PD-L1 or anti-CTLA-4 antibodies could attenuate MDSC-mediated immunosuppression while restoring T cell function, thereby markedly enhancing the clinical efficacy of immunotherapy. Furthermore, m^6^A-targeted agents can potentially be integrated with conventional chemotherapy or radiotherapy. Although standard chemo- and radiotherapies directly eliminate tumor cells, they may also induce MDSC accumulation, limiting therapeutic efficacy; addition of m^6^A modulators could mitigate this effect and improve overall anti-tumor outcomes. Despite these promising prospects, m^6^A-MDSC-targeted therapies face several challenges. The first is specificity: as a pervasive post-transcriptional regulatory mechanism, modulating m^6^A regulators may inadvertently affect normal hematopoietic and immune cell functions, leading to off-target effects. The second challenge lies in delivery: achieving precise delivery of m^6^A inhibitors or activators to MDSCs within the TME, without systemic distribution, remains a key barrier to clinical translation. Finally, safety and toxicity concerns are critical. Given the involvement of m^6^A modification in metabolism, immunity, and cellular homeostasis, long-term systemic interventions may elicit unforeseen adverse effects. Consequently, developing tissue- or cell type-specific delivery systems, such as nanoparticles or targeted antibody-drug conjugates, as well as identifying more precise molecular targets, will be essential for clinical translation of the m^6^A-MDSC axis. Targeting m^6^A regulators can directly attenuate MDSC-mediated immunosuppression while enhancing the efficacy of existing ICIs and conventional chemo- or radiotherapies. Nonetheless, overcoming challenges related to specificity, delivery, and potential toxicity will require further mechanistic studies and technological innovations.

## 6 Challenges and future perspectives

Despite significant progress in recent years regarding the role of m^6^A modification in regulating MDSC function, several challenges remain, limiting its translation into clinical applications (58). First, the high heterogeneity of MDSCs constitutes a major obstacle. MDSCs consist of two principal subsets: PMN-MDSCs and M-MDSCs, which differ in phenotype, molecular mechanisms, and immunosuppressive capacity (81). Most current studies focus on the bulk MDSC population, lacking a detailed analysis of m^6^A modification patterns and functional distinctions among individual subsets. Furthermore, m^6^A may exert context-specific effects depending on the pathological environment; for example, in certain inflammatory or infectious conditions, m^6^A regulation may confer protective effects on MDSCs, whereas in tumors it predominantly promotes immunosuppression (8). Such context-dependency further complicates mechanistic interpretation and clinical translation. Second, technical limitations present another significant bottleneck. While high-throughput sequencing and MeRIP-seq have greatly advanced m^6^A research, these methods primarily operate at the population level and cannot resolve the heterogeneity within MDSCs. Emerging single-cell m^6^A sequencing technologies show promise but still face limitations in sensitivity, specificity, and reproducibility, making them insufficient to fully capture the dynamic landscape of m^6^A modifications within MDSCs. Therefore, the development of more precise, high-resolution single-cell epitranscriptomic approaches represents a key direction for future research. From a clinical translation perspective, targeting the m^6^A-MDSC axis faces multiple challenges. Drug safety is a primary concern, as m^6^A modification is a ubiquitous post-transcriptional regulatory mechanism involved in normal hematopoiesis, metabolism, and immune homeostasis; excessive or prolonged inhibition may lead to severe off-target effects (51). Additionally, patient heterogeneity and molecular subtypes have not been fully considered, as the role of m^6^A in regulating MDSCs may vary significantly across different patients or tumor types, posing challenges for broad therapeutic applicability. The lack of reliable biomarkers also limits patient stratification and efficacy prediction for m^6^A-targeted therapies; developing molecular markers based on m^6^A signatures or MDSC functional states will be critical to facilitate clinical implementation.

Looking forward, research should focus on several directions. First, precise targeting of MDSC subsets is essential. By elucidating subset-specific m^6^A modification patterns, more selective intervention strategies can be developed. Second, combination therapy strategies merit further exploration. Integrating m^6^A-targeted agents with immune checkpoint inhibitors (e.g., anti-[9]PD-1/PD-L1 or anti-CTLA-4) or conventional chemo- and radiotherapy could attenuate MDSC-mediated immunosuppression while enhancing T cell activity, thereby improving the overall response to cancer immunotherapy. Finally, advances in single-cell multi-omics, integrated multi-omics, and artificial intelligence-based analyses are expected to comprehensively map the dynamic regulatory networks of m^6^A in MDSCs, facilitating more precise and personalized immunotherapeutic strategies. In summary, m^6^A modification, as a crucial regulator of MDSC function, is emerging as a frontier in tumor immunotherapy research. Despite challenges related to heterogeneity, technical limitations, and translational hurdles, ongoing advances in basic research and technology are likely to enable the m^6^A–MDSC axis as a novel avenue for cancer immunotherapy.

## 7 Conclusion

m^6^A modification play critical roles in the differentiation, function, and homeostasis of various immune cells. A growing body of studies suggests that m^6^A also serves as a key regulator of the expansion, differentiation, and immunosuppressive activity of MDSCs. By modulating MDSC metabolic pathways, the expression of immunosuppressive factors, and interactions with T cells, dendritic cells, and other immune effector cells, m^6^A not only shapes the tumor immune microenvironment but also profoundly influences anti-tumor immune responses and the efficacy of immunotherapy. Therefore, the m6A-MDSC axis represents a promising molecular target and therapeutic strategy in cancer immunotherapy. Looking forward, with the continuous advancement of single-cell multi-omics and epitranscriptomic technologies, the subset-specific roles of m^6^A in MDSCs are expected to be elucidated in greater detail. This will help clarify the context-dependent effects of m^6^A under different pathological conditions and facilitate the development of personalized therapeutic strategies. Furthermore, combining m^6^A-targeted agents with immune checkpoint inhibitors, radiotherapy, or chemotherapy holds the potential to modulate the immune microenvironment on multiple fronts, thereby enhancing the clinical efficacy of cancer immunotherapy. Although challenges related to drug specificity, delivery systems, and safety remain, it is foreseeable that, as our understanding of the m^6^A-MDSC axis deepens, this approach will offer broad applications in the field of tumor immunotherapy.
